# Cellulitis or Lymphadenopathy: A Challenging Monkeypox Virus Infection Case

**DOI:** 10.7759/cureus.40008

**Published:** 2023-06-05

**Authors:** Mohammed H Hussein, Muna A Mohamad, Sneha Dhakal, Monica Sharma

**Affiliations:** 1 Internal Medicine, Ascension Saint Joseph Hospital, Chicago, USA; 2 Infectious Disease, Ascension Saint Joseph Hospital, Chicago, USA

**Keywords:** lymphadenopathy, submandibular, submental, cellulitis, monkeypox

## Abstract

Monkeypox virus infection is characterized by a prodromal illness with fever, intense headache, lymphadenopathy, back pain, myalgias, and asthenia, followed by the eruption of skin lesions. A case series has reported monkeypox virus infection with primary anogenital and facial cellulitis. In addition, superimposed bacterial infections have been reported in several case reports. We present a monkeypox virus infection case of a patient presenting with jaw swelling initially thought to be secondary to cellulitis/abscess collection.

A 25-year-old homosexual male on HIV pre-exposure prophylaxis presented to an urgent care center with a painful, ruptured, crusted chin lesion. Given recent contact with monkeypox virus-infected patients, a monkeypox swab was collected. He then developed a fever, jaw/neck swelling, and difficulty swallowing, which prompted him to come to our emergency department. He was febrile and tachycardic on presentation. The labs were unremarkable. A CT scan of the neck showed soft tissue thickening within the submental and submandibular regions bilaterally, consistent with cellulitis without evidence of abscess formation. It also showed prominent bilateral submandibular and left station IIA lymphadenopathy. We started the patient on intravenous ampicillin-sulbactam, but his swelling worsened. We suspected abscess formation clinically; however, a percutaneous drainage attempt yielded a dry tap. We added vancomycin for extra coverage, but the patient remained febrile, and his swelling continued to worsen. In the meantime, his monkeypox virus polymerase chain reaction (PCR) swab result returned positive, and he developed other skin lesions. These two findings and the lack of improvement with antibiotic therapy led us to believe that his fever was secondary to monkeypox and the swelling was secondary to reactive lymphadenopathy over true cellulitis. We stopped his antibiotics, and his symptoms improved with a complete resolution of the jaw swelling.

This case was challenging to manage as the patient’s swelling was initially thought to be secondary to cellulitis and abscess collection, but it turned out to be secondary to lymphadenopathy. This case illustrates the significance and severity of lymphadenopathy in monkeypox virus infection, which can be initially misdiagnosed as cellulitis.

## Introduction

Monkeypox virus is an enveloped double-stranded DNA virus that belongs to the Orthopox genus of the Poxviridae family. Previously, this virus was rarely reported outside of western and central Africa. However, in May 2022, multiple cases of monkeypox virus infection were identified in several non-endemic countries, including the United States [1].

The infection is characterized by a prodromal illness with fever, intense headache, lymphadenopathy, back pain, myalgia, and asthenia [1]. The skin eruption usually begins within one to three days of the onset of the fever [1]. Lymphadenopathy is a distinctive feature of monkeypox compared to other diseases that may initially appear similar (e.g., chickenpox, measles, and smallpox) [2].

Superimposed bacterial infection of the lesions has been reported in several case reports and case series [3-5]. We report a monkeypox virus infection in a 25-year-old male who presented with submental swelling initially treated as primary bacterial cellulitis and a skin abscess.

This article was previously presented as an oral clinical vignette at the 2022 ACP Illinois Chapter's Oral Clinical Vignette, Story Slam, and Art in Medicine on November 16, 2022.

## Case presentation

A 25-year-old homosexual male on HIV pre-exposure prophylaxis presented to an urgent care center two days after developing a painful lesion on his chin. The lesion progressively increased in size and then ruptured. At the urgent care center, swabs from the lesion were collected for the orthopox and monkeypox virus polymerase chain reaction (PCR). The patient received the monkeypox vaccine at the urgent care center as he reported recent non-sexual close contact with monkeypox virus-exposed individuals. Six days later, he started having fever spikes, myalgia, and diffuse painful jaw swelling, causing difficulty swallowing, which prompted him to revisit the urgent care center. He was prescribed topical mupirocin, oral clindamycin, and oral valacyclovir. Unfortunately, the patient's fever worsened, so he presented to our emergency department (ED) the following day.

**Figure 1:**
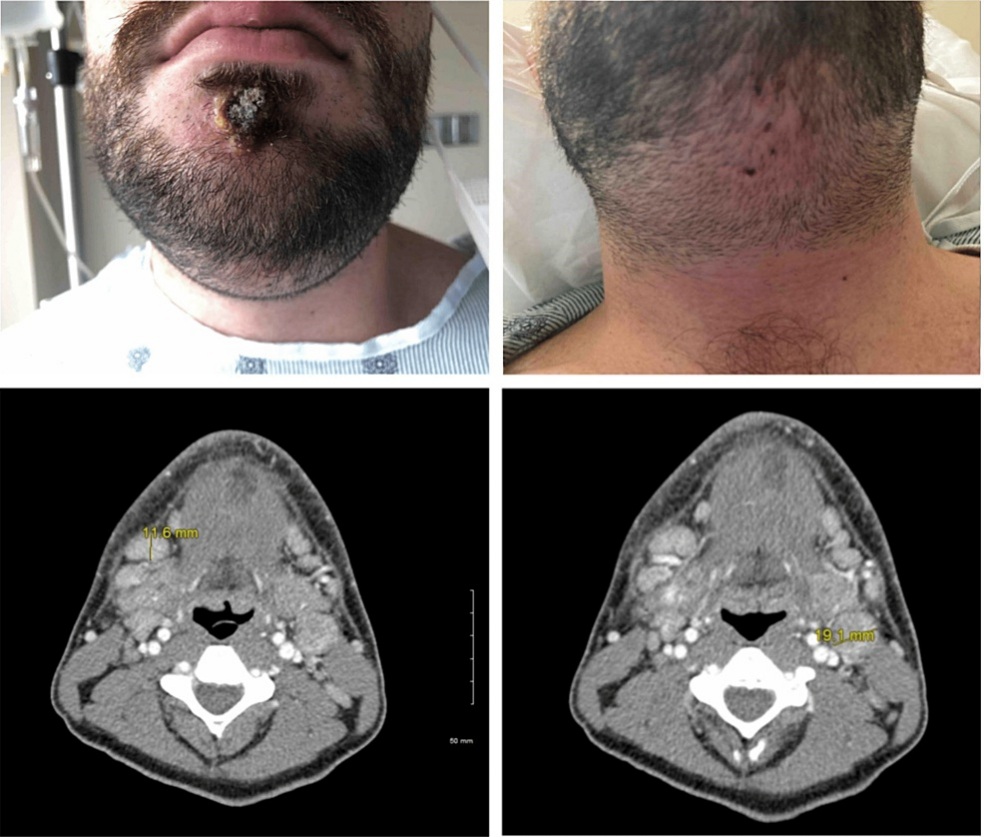
The clinical and radiographic findings of the patient's jaw swelling Left upper: A crusted 3 cm × 3 cm lesion on the patient's chin. Right upper: Diffuse swelling and erythema at the patient's jaw/neck area. Left lower: Enlarged right submandibular lymph node measuring 1.2 cm on the axial view of the patient's CT neck. Right lower: Enlarged left station IIA lymph node measuring 1.9 cm on the axial view of the patient's CT neck.

In the ED, he was febrile with a temperature of 100.5 °F, had a heart rate of 130 beats per minute, and had a blood pressure of 134/78. On physical examination, there was diffuse swelling, erythema, tenderness, and induration of the submental region, suspicious of abscess collection (Figure 1). In addition, a 3 cm × 3 cm crusted and ruptured pustule was noted on his chin (Figure 1). Laboratory results revealed a white blood cell count of 5,500 cells/µl and hemoglobin of 15.0 g/dL. The patient's renal function and electrolytes were within normal range. Monkeypox swab results were still pending at that time. An enhanced axial neck CT scan with intravenous contrast revealed soft tissue thickening within the submental and submandibular regions bilaterally, consistent with cellulitis (Figure 1). It also showed prominent bilateral submandibular and left station IIA lymphadenopathy. The right submandibular lymphadenopathy was 1.2 cm in diameter, and the left station IIA was 1.9 cm (Figure 1). However, there was no focal rim-enhancing fluid collection to indicate an abscess.

Therefore, we treated the patient empirically for bacterial cellulitis with intravenous ampicillin-sulbactam as per the advice of the infectious diseases team. On day 2 of the hospitalization, his swelling worsened and became more indurated. We suspected abscess formation; hence, we consulted the otolaryngologist to drain it. He attempted needle aspiration of the swelling at multiple sites with no fluid return. We then added vancomycin to the antibiotic coverage per the otolaryngologist's recommendations and the infectious diseases specialist's approval.

On day 3, the patient's jaw swelling worsened with prominent bilateral cervical lymphadenopathy. Monkeypox viral DNA PCR resulted as positive. Despite 48 hours of intravenous antibiotics, the patient continued to spike fevers and developed three other pustular lesions on his left posterior thigh, elbow, and right ankle. We thought monkeypox could explain his fever, and we concluded that his swelling was likely reactive lymphadenopathy rather than cellulitis. Considering this, we withheld the antibiotics and observed him for 24 hours off antibiotics. He received supportive treatment and analgesia. Subsequently, there was a notable improvement in the swelling, and he was discharged home on day 5. Antiviral therapy was not indicated as there was no ocular involvement or painful mucosal lesions, and the patient was not immunocompromised.

In a one-week follow-up, the patient reported doing well with complete neck and jaw swelling resolution. In addition, he denied the appearance of any other lesions.

## Discussion

Monkeypox virus is a zoonotic orthopox DNA virus first described in humans in the Democratic Republic of the Congo in 1970. Since then, sporadic outbreaks of this disease in Africa have been attributed to contact with wild animals (mainly rodents). Human-to-human spread was noticed around May 2022, resulting in outbreaks in the Western world. The mechanism of human-to-human spread was found to be secondary to large respiratory droplets and direct or close contact with the skin lesions. Sexual transmission has not been confirmed. However, a significantly higher proportion of infections was observed in men who are gay, bisexual, or have sex with other men. [3]

The infection is characterized by an incubation period of 5-21 days, followed by a prodromal illness with fever, intense headache, lymphadenopathy, back pain, myalgia, and asthenia [1,2]. Then, skin eruptions begin within one to three days of the onset of the fever [1]. The disease course consists of viral inoculation and replication in the oropharyngeal and respiratory mucosa, then primary viremia and spread to lymphoid organs, followed by secondary viremia to the skin and tertiary organs [6]. However, lymphadenopathy is a distinctive feature of monkeypox virus infection compared to other diseases that may initially appear similar (e.g., chickenpox, measles, and smallpox) [2].

In an observational analysis of confirmed monkeypox cases in the United Kingdom (UK), all patients presented with skin lesions, of which 94% were anogenital [7]. This analysis also reported cases presenting with facial and anogenital cellulitis rather than distinct skin lesions [7]. There have been other case series and reports of patients presenting with cellulitis as a secondary infectious complication of monkeypox rather than a primary presentation [3-5].

The diagnosis is made by PCR testing of the lesions, with the highest yield of positive results when the swab is obtained from the skin and anogenital lesions [3]. Most monkeypox virus cases are self-limiting. Antiviral therapy (Tecovirimat) is preserved for patients with severe disease (e.g., encephalitis, hemorrhagic rash, etc.), patients at high risk of developing severe illness (e.g., immunocompromised, pregnant women, ocular lesions, etc.), and patients with an increased risk of spreading the disease (e.g., anogenital lesions, etc.). In the United States, vaccination against the disease is recommended for those 18 years of age or older who are at high risk of contracting the virus [8].

Given our patient's fever, pain, and difficulty swallowing, we initially thought our patient had submental and submandibular cellulitis and an abscess collection from the pustular lesion on his chin. Our clinical suspicion for monkeypox was low, given there were no anogenital or oral lesions in addition to the distinct submental induration and erythema on physical exam. Even though contact and droplet isolation were maintained, we treated the patient empirically for bacterial cellulitis and abscess collection with intravenous antibiotics and an abscess drainage attempt, respectively. The dry tap of the swelling ruled out abscess collection, but we were still treating the patient for bacterial cellulitis.

However, the persistent fever and the worsening of the swelling pointed toward antibiotic treatment failure. These two findings, in addition to the positive monkeypox PCR result and the appearance of new lesions, led us to think that the indurated swelling was likely due to enlarged lymph nodes from monkeypox rather than bacterial cellulitis. Furthermore, the improvement of the swelling off-antibiotics supported our thought.

## Conclusions

Soft tissue swelling in the setting of monkeypox virus infection can be challenging to differentiate as it can be reactive lymphadenopathy or a concomitant/superimposed bacterial cellulitis. Considering reactive lymphadenopathy as a potential cause of soft tissue swelling in the proper clinical context will reduce the need for unnecessary antibiotics in monkeypox patients.
